# The disease burden of ILD at the global, regional, and national levels from 1990 to 2021, and projections until 2035: the 2021 Global Burden of Disease study

**DOI:** 10.3389/fmed.2025.1637654

**Published:** 2025-08-13

**Authors:** Lili Zhang, Junyi Zhang, Yikun Guo, Jun Yan

**Affiliations:** ^1^Dongzhimen Hospital, Beijing University of Chinese Medicine, Beijing, China; ^2^Beijing University of Chinese Medicine, Beijing, China; ^3^Department of Pulmonary, Beijing University of Chinese Medicine Shenzhen Hospital (Longgang), Shenzhen, China

**Keywords:** interstitial lung disease, incidence rate mortality rate, disability life adjustment year, Joinpoint regression model, Bayesian age period queue model

## Abstract

**Background:**

Interstitial lung disease (ILD) is an important component of the global disease burden and health challenges. This study aims to describe and analyze the global and regional disease burden of ILD, and predict future epidemiological trends.

**Methods:**

The data from the 2021 Global Burden of Disease, Injury and Risk Factors (GBD) were used to conduct a secondary analysis on the incidence rate, mortality and disability adjusted life year (DALY) of ILD in 21 regions and 204 countries and regions from 1990 to 2021, stratified by time, space, gender, age and social demographic index (SDI). The Joinpoint regression model is used to calculate the average annual percentage change (AAPC). Using Bayesian Age Period cohort (BAPC) model to predict the global disease burden trend of ILD in 2035.

**Results:**

In 2021, the incidence, mortality, and DALY of ILD have all increased compared to before, while the ASIR, ASMR, and ASDR of ILD have also shown an upward trend. It is worth noting that the disease burden of males and the elderly is generally higher than that of females and other age groups. Meanwhile, cross-border inequality analysis shows that a disproportionate burden of ILD is concentrated in countries with higher SDI. Decomposition analysis shows that population growth and aging have become important influencing factors of ILD disease burden. The age period cohort effect indicates a roughly positive correlation between disease risk and age. Frontier analysis indicates that countries with higher SDI have greater potential for burden improvement. It is expected that the disease burden of ILD will increase by 2035.

**Conclusion:**

The disease burden of ILD remains an important health issue globally. Exploring disease differences among different regions, genders, and ages, analyzing the disease burden and future trends of ILD, is of great significance for developing targeted prevention strategies, optimizing healthcare accessibility, and improving diagnostic interventions.

## 1 Introduction

ILD is a general term for a series of heterogeneous lung diseases, characterized by various forms of chronic inflammation or fibrosis in the interstitium and alveoli of the lungs, affecting gas exchange in the lungs (1), causing difficulty breathing, decreased activity tolerance, and ultimately leading to respiratory failure or even death (2, 3). The diagnosis of ILD relies on detailed medical history collection and high-resolution computed tomography (HRCT); at the same time, the therapeutic effects of different subtypes of ILD vary, and lung transplantation remains the only curative therapy in the late stages of the disease (4). Therefore, the diagnosis and intervention of ILD remain a complex and challenging task (5, 6).

Previous epidemiological studies have mostly been limited to subtypes of ILD diseases, such as systemic sclerosis related interstitial lung disease (7), rheumatoid arthritis interstitial lung disease (8), smoking related interstitial lung disease (9), connective tissue related interstitial lung disease (10), etc.; or concentrated in a certain group, such as children with interstitial lung disease (11) or familial interstitial lung disease (12). In addition, GBD ILD 2021 shows that the disease burden of ILD has been increasing worldwide (13). However, this study focused on evaluating the independent effects of age, period, and cohort on the burden of ILD disease, without conducting a comprehensive and in-depth analysis in conjunction with other novel analytical methods. Yang (14) et al. focused on evaluating the incidence trend of ILD in BRICS countries from 1990 to 2021. Although they provided data support for the formulation of public health policies in these countries, there are significant socio-economic and cultural differences within BRICS countries themselves, which may mask specific disease problems or incidence characteristics of certain countries.

This study obtained data related to ILD from the latest Global Burden of Disease (GBD) 2021 report, which provides important foundational information for our research. By analyzing stratification factors such as gender, age, and demographic indices, we have gained a better understanding of the distribution of diseases among different groups. In addition, we have also adopted some new research methods, such as health inequality analysis, decomposition analysis, and frontier analysis, as well as various epidemiological indicators, such as age period cohort models and Bayesian prediction models, to interpret the changing trends of ILD from the perspectives of time and space, providing important references and guidance for formulating public health strategies, promoting international cooperation, and improving diagnosis and treatment technologies (15).

## 2 Materials and methods

### 2.1 Data sources

The latest GBD database released in 2024 analyzed 100,983 data sources from 204 countries and regions between 1990 and 2021, covering 371 diseases, injuries, and 88 risk factors. Its original data source is obtained through http://ghdx.healthdata.org/gbd-results-tool Online queries, including various datasets such as population census, household survey, civil registration, vital statistics, disease notification, health service contact, air pollution monitoring, and satellite imaging (16). This database ensures internal consistency of data across different regions, ages, genders, and years through system adjustments (17). This study followed the Guidelines for Accurate and Transparent Health Estimation Reporting (GATHER) (18) (Supplementary Table S1).

### 2.2 Definition

Interstitial lung diseases can be roughly divided into idiopathic, autoimmune disease-related, environmental or occupational exposure related (including iatrogenic), interstitial lung diseases with cysts or air filled cavities, sarcoidosis, and unexplained interstitial lung diseases (1). The severity of the disease ranges from insidious to acute, and even life-threatening. GBD 2021 uses the definition of the International Classification of Diseases and Injuries (ICD) diagnostic code to estimate and analyze disease burden outcomes. The codes for ILD in the 10th edition of the International Classification of Diseases (ICD-10) are J84 and D86.

In the Global Burden of Disease (GBD) database, the Social Development Index (SDI) is a comprehensive indicator that measures the socio-economic development level of a country or region. Its calculation takes into account multiple factors, including average household or individual income, adult education level, and total fertility rate (TFR) per thousand women (19) per thousand women. The geometric mean of three indices provides the numerical values of SDI at the national level, divided into five groups: low SDI (SDI < 0.45), low middle SDI (0.45 ≤ SDI < 0.61), middle SDI (0.61 ≤ SDI < 0.69), high middle SDI (0.69 ≤ SDI < 0.80), and high SDI (SDI ≥ 0.80) (20).

### 2.3 Data statistics

Estimated Annual Percentage Change (EAPC) is used to analyze the trend of a disease burden or health indicator over consecutive years, with a 95% confidence interval (CI) obtained from a linear regression model (21). The Joinpoint regression model can identify trend change points in data and estimate the slope of each trend segment, thereby more accurately describing the overall trend of the time series. Compared to traditional regression models, the Joinpoint model uses the rule of minimizing the sum of squared residuals to estimate the change in disease burden, avoiding the non-objectivity based on typical linear trend analysis, and using the Average Annual Percentage Change (AAPC) to represent the results (22), and the statistical significance of the trend is determined by the 95% confidence interval (CI). The study suggests that *p* < 0.05 has statistical significance. All statistical analyses and data visualizations were performed using R (version 4.4.2) and JD_GBDR (V2.37, Jingding Medical Technology Co., Ltd.). In this study, the R software package (version 4.2.3) and JD_GBDR (V2.22, Jingding Medical Technology Co., Ltd.) was used for the drawing of the figures.

### 2.4 Analysis methods

#### 2.4.1 Decomposition analysis

Decomposition Analysis (DA) is the use of Das Gupta’s method to decompose the burden changes of ILD into population aging, population growth, and epidemiological changes, and quantify the contribution of each factor to the overall changes (23). By using decomposition analysis to study the epidemiology of ILD disease, the degree of influence of various driving factors, and the effectiveness of different intervention measures, we can not only better understand the essence of these issues, but also identify the areas that need to be focused on in public health, so as to implement targeted measures.

#### 2.4.2 Analysis of health inequality

Health inequality refers to the differences in health status, access to medical services, and health outcomes among different social groups (24). The Slope Index of Inequality (SII) and Concentration Index (CI) are important tools for measuring and evaluating health inequality (25). The slope index is calculated through regression analysis, representing the absolute difference in health outcomes between the lowest and highest levels of socioeconomic status distribution. The concentration index reflects the degree of concentration of health outcomes in socioeconomic status. Usually calculated through Lorenz Curve and Concentration Curve (26).

#### 2.4.3 Frontier analysis

Frontier Analysis is used to evaluate the progress and gaps of countries in achieving specific health goals. Firstly, establish a “best practice” or “cutting-edge” standard, which is typically based on actual data from the best performing countries or regions. Then, by comparing data from other countries or regions with this “cutting-edge” standard, evaluate their performance in achieving specific health goals (17). Frontier analysis can clearly demonstrate the progress and gaps in achieving specific health goals in various countries or regions, thereby formulating more targeted intervention measures, promoting policy improvement, and contributing to achieving global health equity.

#### 2.4.4 Age period cohort analysis

We divided the time dimension from 1990 to 2021 into six consecutive time periods and divided them into 20 consecutive cohorts with a 5-year-old interval to analyze the differences in ILD disease burden among different age groups, time periods, and birth cohorts. The net drift represents the change rate of the overall trend of the incidence rate, mortality and DALY rate of ILD, that is, the long-term change trend of health outcomes over time during the entire study period; And local drift refers to the short-term rate of change within a specific time period, that is, the trend of changes in ILD disease burden during a specific period (27). Both can comprehensively understand the multidimensional influencing factors of health outcomes, evaluate long-term and short-term health trends, and provide strong support for policy formulation and public health interventions.

#### 2.4.5 Bayesian prediction model

The Bayesian age period queue model (28)uses an integrated nested Laplacian approximation and assumes the inverse gamma prior distribution of existing data, providing a practical method for predicting ILD disease burden. The structure of this model is a logarithmic linear Poisson model, which assumes the multiplication effect of age, period, and queue variables, and is implemented using R-BAPC and R-INLA software packages (29). By introducing prior distributions, these uncertainties can be effectively handled, improving the reliability of estimates. In addition, Bayesian models can also predict future trends based on historical data, playing a key role in GBD research. They not only improve the efficiency and accuracy of data utilization, but also provide strong support for the formulation of global public health strategies.

## 3 Results

### 3.1 Overview of disease burden of ILD

**Figure 1:**
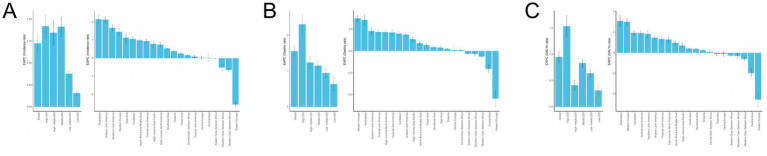
EAPC analysis of ILD. **(A)** ASIR; **(B)** ASMR; **(C)** ASDR.

Worldwide, the number of incidence for ILD in 2021 was 390,267 people (Table 1), an increase of 147.88% compared to 1990; ASIR for ILD increased from 3.767 (95% UI: 3.268–4.277) per 100,000 in 1990 to 4.545 (95% UI: 4.055–5.038) per 100,000 in 2021, and the EAPC was 0.73 (95% CI: 0.63 to 0.82) (Figure 1). From 1990 to 2021, the number of death for ILD increased from 54,967 people to 188,222 people; ASMR for ILD increased from 1.52 (95% UI: 1.25–1.87) per 100,000 to 2.28 (95% UI: 1.96–2.56) per 100,000, with an EAPC of 1.553 (95% CI: 1.410 to 1.697). The number of DALYs caused by ILD is continuously increasing worldwide, with 4,042,150 people in ILD in 2021, an increase of 169.29% compared to 1990; ASDR for ILD increased from 37.148 per 100,000 in 1990 (95% UI: 30.617–45.366) to 47.618 per 100,000 in 2021 (95% UI: 41.258–53.165), with an EAPC of 0.952 (95% CI: −0.851 to 1.053).

**Table 1:** The case number and ASR of ILD in 1990 and 2021.

GBD data		Incidence					Deaths					DALYs				
		1990		2021		EAPC (1990–2021) (95% CI)	1990		2021		EAPC (1990–2021) (95% CI)	1990		2021		EAPC (1990–2021) (95% CI)
		All ages (numbers)	Age-standardized rate (per 100,000 people)	All ages (numbers)	Age-standardized rate (per 100,000 people)		All ages (numbers)	Age-standardized rate (per 100,000 people)	All ages (numbers)	Age-standardized rate (per 100,000 people)		All ages (numbers)	Age-standardized rate (per 100,000 people)	All ages (numbers)	Age-standardized rate (per 100,000 people)	
Global																
	Both	157441.174 (136251.286, 179471.825)	3.767 (3.268, 4.277)	390267.107 (346393.415, 433403.270)	4.545 (4.055, 5.038)	0.725 (0.626, 0.824)	54967.234 (44761.390, 68391.194)	1.517 (1.251, 1.868)	188222.370 (161405.656, 212251.519)	2.280 (1.959, 2.563)	1.553 (1.410, 1.697)	1501028.432 (1221196.884, 1850556.940)	37.148 (30.617, 45.366)	4042150.489 (3489794.642, 4516882.923)	47.618 (41.258, 53.165)	0.952 (0.851, 1.053)
	Male	86263.966 (74477.206, 98468.219)	4.481 (3.892, 5.051)	214681.179 (190533.195, 238498.186)	5.365 (4.802, 5.953)	0.707 (0.596, 0.818)	31182.606 (24196.086, 39011.603)	2.010 (1.606, 2.481)	103056.700 (84156.395, 115833.397)	2.899 (2.397, 3.238)	1.407 (1.279, 1.535)	853132.962 (666666.893, 1066356.171)	46.484 (36.620, 57.591)	2237269.367 (1839499.938, 2555199.732)	57.788 (47.499, 65.767)	0.850 (0.755, 0.946)
	Female	71177.208 (61733.290, 81297.246)	3.226 (2.805, 3.678)	175585.928 (155725.247, 195606.717)	3.886 (3.456, 4.313)	0.714 (0.628, 0.801)	23784.628 (18180.453, 32746.567)	1.168 (0.902, 1.599)	85165.669 (68720.001, 105539.291)	1.827 (1.475, 2.265)	1.698 (1.533, 1.864)	647895.470 (500164.802, 883303.445)	29.787 (23.229, 40.191)	1804881.122 (1465706.809, 2216375.571)	39.486 (31.952, 48.620)	1.065 (0.957, 1.173)
SDI quintiles + GBD regions																
	East Asia	19462.899 (16280.977, 23141.040)	1.898 (1.605, 2.227)	50030.893 (42927.494, 57599.826)	2.313 (2.022, 2.639)	1.122 (0.854, 1.391)	3042.053 (2259.018, 4830.813)	0.407 (0.309, 0.657)	8189.678 (5177.143, 10926.658)	0.405 (0.255, 0.538)	0.383 (0.190, 0.576)	115445.088 (86137.562, 171273.594)	12.409 (9.415, 18.310)	234265.799 (171304.550, 301444.133)	11.015 (8.056, 14.138)	−0.123 (−0.281, 0.034)
	Oceania	155.305 (138.585, 173.867)	3.717 (3.373, 4.087)	419.296 (384.884, 456.213)	4.200 (3.906, 4.509)	0.385 (0.360, 0.410)	71.236 (47.875, 109.639)	2.160 (1.448, 3.464)	185.382 (118.672, 304.185)	2.298 (1.416, 3.847)	0.231 (0.163, 0.298)	3218.843 (2152.507, 4772.886)	69.387 (47.612, 104.633)	8024.271 (5581.369, 12403.398)	74.630 (50.209, 118.430)	0.250 (0.169, 0.331)
	Southeast Asia	3838.789 (3217.544, 4512.250)	1.360 (1.164, 1.564)	11245.442 (9856.839, 12737.719)	1.617 (1.424, 1.809)	0.545 (0.531, 0.559)	658.716 (317.292, 1480.206)	0.292 (0.143, 0.655)	1876.583 (958.525, 3772.331)	0.330 (0.170, 0.655)	0.422 (0.341, 0.504)	22494.859 (12086.731, 47186.405)	8.006 (4.354, 16.313)	59047.923 (34100.112, 109401.612)	8.930 (5.184, 16.515)	0.372 (0.317, 0.426)
	Central Asia	1672.438 (1504.387, 1851.106)	3.358 (3.055, 3.698)	2848.587 (2621.602, 3093.582)	3.380 (3.124, 3.647)	0.016 (−0.212, 0.245)	714.983 (616.274, 821.266)	1.639 (1.378, 1.910)	629.811 (514.538, 778.720)	0.866 (0.711, 1.061)	−2.054 (−2.509, -1.597)	20131.183 (18089.073, 22762.621)	40.302 (35.894, 45.693)	19790.214 (16339.433, 24518.198)	23.371 (19.372, 28.781)	−1.984 (−2.396, -1.571)
	Eastern Europe	5321.508 (4509.716, 6209.125)	2.070 (1.781, 2.401)	2682.316 (2299.151, 3096.100)	1.040 (0.889, 1.211)	−2.563 (−2.681, -2.445)	2860.223 (2659.609, 3059.910)	1.101 (1.021, 1.177)	1153.551 (1054.151, 1263.434)	0.335 (0.307, 0.367)	−5.474 (−6.401, -4.538)	79278.830 (72135.692, 86326.293)	29.379 (26.781, 31.975)	34251.587 (31150.631, 37997.434)	10.784 (9.778, 11.996)	−4.556 (−5.262, -3.844)
	Central Europe	3649.645 (3250.810, 4100.121)	2.585 (2.299, 2.905)	4033.559 (3661.378, 4442.546)	2.425 (2.201, 2.687)	0.016 (−0.072, 0.105)	1513.163 (1419.939, 1622.206)	1.043 (0.982, 1.118)	2179.080 (1973.342, 2369.327)	0.974 (0.884, 1.059)	0.121 (−0.222, 0.465)	44911.609 (41926.367, 48292.808)	30.888 (28.892, 33.117)	53275.237 (48498.569, 58218.189)	26.900 (24.412, 29.448)	−0.126 (−0.377, 0.127)
	Australasia	798.391 (716.650, 884.742)	3.429 (3.078, 3.798)	3392.344 (3070.027, 3718.022)	6.446 (5.879, 7.020)	2.150 (1.926, 2.373)	263.423 (241.612, 284.768)	1.131 (1.030, 1.223)	1903.776 (1602.544, 2087.524)	3.170 (2.686, 3.456)	3.545 (2.979, 4.114)	5708.105 (5219.076, 6189.998)	24.116 (22.114, 26.101)	33035.994 (28955.568, 35886.009)	59.970 (53.126, 64.898)	3.096 (2.535, 3.661)
	Southern Latin America	2749.806 (2529.382, 2974.594)	5.988 (5.513, 6.474)	8476.900 (7913.382, 9062.319)	9.904 (9.260, 10.572)	1.676 (1.527, 1.826)	1140.167 (1066.428, 1222.720)	0.910 (0.837, 1.019)	4356.529 (3905.664, 4680.941)	1.192 (1.041, 1.313)	2.293 (1.902, 2.686)	27988.579 (26106.436, 29940.292)	60.448 (56.322, 64.625)	86238.549 (79827.812, 92166.393)	98.578 (91.539, 105.256)	1.822 (1.514, 2.131)
	High-income Asia Pacific	18818.053 (16024.169, 22063.872)	9.151 (7.819, 10.656)	43787.314 (38399.138, 49649.777)	11.595 (10.250, 13.023)	0.787 (0.595, 0.978)	5195.838 (4709.077, 5607.992)	2.718 (2.450, 2.936)	26166.439 (21825.749, 28825.677)	4.356 (3.726, 4.761)	1.346 (1.107, 1.586)	132227.157 (120398.344, 145561.199)	66.255 (60.195, 72.902)	432702.509 (380225.136, 475662.826)	86.318 (76.637, 94.927)	0.705 (0.510, 0.901)
	Central Sub-Saharan Africa	456.176 (384.881, 531.222)	1.797 (1.547, 2.053)	1271.704 (1103.243, 1454.641)	1.879 (1.662, 2.097)	0.166 (0.130, 0.203)	258.944 (87.555, 548.896)	1.417 (0.487, 3.368)	665.971 (241.527, 1621.743)	1.472 (0.508, 3.917)	0.091 (−0.017, 0.199)	8097.075 (2924.875, 15523.969)	32.706 (12.315, 68.731)	20544.531 (8466.675, 44098.944)	33.845 (13.174, 79.520)	0.085 (−0.019, 0.189)
	Tropical Latin America	2580.472 (2183.944, 3040.080)	2.436 (2.083, 2.820)	6639.974 (5821.179, 7441.328)	2.617 (2.291, 2.941)	0.089 (0.018, 0.161)	846.584 (799.384, 890.235)	2.081 (1.075, 3.246)	4527.093 (4080.055, 4835.634)	1.995 (1.390, 2.649)	2.130 (1.790, 2.472)	27651.278 (26178.367, 29362.867)	26.406 (24.856, 28.109)	102784.236 (95498.424, 108206.434)	40.581 (37.645, 42.740)	1.330 (1.047, 1.615)
	Andean Latin America	2439.725 (2227.637, 2661.719)	12.293 (11.132, 13.456)	11837.689 (11093.939, 12531.267)	20.467 (19.150, 21.686)	2.126 (1.972, 2.281)	1443.633 (1044.936, 2077.775)	1.109 (1.035, 1.158)	6366.227 (4874.545, 8022.423)	2.787 (2.493, 2.972)	1.873 (1.683, 2.062)	33944.989 (24797.829, 48279.533)	158.265 (116.076, 221.595)	121673.133 (96602.283, 150014.430)	209.339 (165.807, 257.665)	1.466 (1.286, 1.646)
	Western Europe	18947.570 (17006.943, 20973.687)	3.644 (3.264, 4.051)	43619.932 (39614.115, 47748.761)	5.298 (4.820, 5.807)	1.456 (1.282, 1.630)	6644.622 (6203.761, 6943.690)	7.859 (5.682, 11.331)	30538.319 (26844.519, 32788.124)	11.374 (8.687, 14.332)	3.742 (3.377, 4.108)	154696.915 (143922.777, 165738.605)	27.507 (25.548, 29.621)	526089.795 (478285.854, 559276.079)	56.372 (51.973, 59.883)	2.998 (2.649, 3.348)
	South Asia	35968.280 (30515.173, 41709.481)	6.005 (5.109, 6.881)	96281.455 (84137.368, 108635.455)	6.439 (5.650, 7.262)	0.249 (0.221, 0.276)	17522.095 (9598.192, 27912.600)	3.616 (2.004, 5.648)	54724.110 (35863.108, 74242.874)	4.192 (2.812, 5.764)	0.668 (0.536, 0.800)	473027.602 (268377.064, 740982.721)	81.676 (47.160, 126.638)	1312643.600 (890805.920, 1740638.785)	89.796 (61.006, 118.919)	0.402 (0.332, 0.472)
	High-income North America	28333.491 (24529.709, 32456.438)	8.483 (7.369, 9.706)	66609.122 (58422.822, 75152.764)	10.954 (9.727, 12.202)	0.749 (0.617, 0.882)	8065.652 (7441.856, 8420.527)	2.233 (2.068, 2.328)	29737.312 (26085.079, 31529.351)	4.246 (3.752, 4.486)	2.164 (1.797, 2.533)	210856.165 (195639.685, 227582.521)	61.652 (57.124, 66.567)	582574.850 (532853.221, 621774.914)	90.444 (83.276, 96.544)	1.279 (0.989, 1.571)
	Western Sub-Saharan Africa	1436.210 (1203.763, 1682.076)	1.369 (1.172, 1.576)	2980.762 (2544.172, 3485.630)	1.130 (0.992, 1.280)	−0.649 (−0.718, -0.580)	1332.317 (486.023, 2109.004)	1.727 (0.655, 2.771)	2386.495 (919.027, 4115.003)	1.389 (0.540, 2.412)	−0.638 (−0.760, -0.516)	36521.983 (13622.666, 55767.251)	38.641 (15.119, 59.967)	69547.034 (29750.716, 115085.424)	31.333 (13.044, 52.940)	−0.615 (−0.736, -0.494)
	Caribbean	393.607 (343.567, 448.413)	1.416 (1.253, 1.599)	1003.145 (917.012, 1094.448)	1.893 (1.727, 2.072)	1.063 (0.979, 1.147)	174.736 (140.891, 218.992)	0.696 (0.573, 0.860)	639.265 (534.389, 773.276)	1.186 (0.991, 1.447)	1.993 (1.773, 2.213)	5039.731 (3866.338, 6913.750)	18.070 (14.090, 23.622)	15715.280 (12779.325, 19980.939)	29.998 (24.151, 38.597)	1.924 (1.732, 2.116)
	High-middle SDI	25615.727 (22401.259, 29234.518)	2.493 (2.195, 2.833)	56001.459 (50154.737, 61921.277)	2.970 (2.684, 3.266)	0.845 (0.700, 0.991)	8237.681 (7611.498, 9226.601)	0.980 (0.905, 1.044)	22850.827 (20008.186, 25163.440)	1.830 (1.645, 1.959)	1.237 (1.081, 1.393)	237514.378 (213560.478, 266885.043)	23.853 (21.525, 26.810)	485511.717 (430895.703, 541669.294)	25.479 (22.627, 28.472)	0.411 (0.318, 0.505)
	Low-middle SDI	28178.889 (23956.492, 32564.899)	4.392 (3.756, 5.001)	70990.237 (62702.072, 79388.324)	4.849 (4.276, 5.420)	0.378 (0.348, 0.408)	13152.917 (7523.033, 20491.259)	2.483 (1.455, 3.808)	39117.855 (26539.668, 53611.717)	3.085 (2.124, 4.228)	0.943 (0.825, 1.061)	359489.057 (213851.924, 552928.685)	57.351 (34.674, 86.875)	949437.881 (662365.395, 1260418.622)	66.549 (46.338, 88.364)	0.637 (0.567, 0.708)
	Eastern Sub-Saharan Africa	1327.803 (1107.276, 1558.830)	1.522 (1.306, 1.739)	3243.789 (2779.589, 3752.945)	1.528 (1.344, 1.724)	−0.018 (−0.031, -0.006)	686.513 (228.307, 1205.907)	1.025 (0.352, 1.934)	1483.871 (530.270, 3097.873)	0.961 (0.333, 2.082)	−0.317 (−0.375, -0.259)	22391.044 (7514.983, 36231.901)	24.768 (9.296, 43.148)	47826.032 (19641.778, 93157.054)	23.453 (9.359, 46.538)	−0.289 (−0.341, -0.236)
	North Africa and Middle East	4326.186 (3719.778, 5016.180)	2.189 (1.906, 2.488)	15113.488 (13573.573, 16830.538)	2.853 (2.578, 3.151)	0.980 (0.924, 1.037)	919.068 (633.261, 1433.163)	2.579 (2.408, 2.767)	3051.397 (2249.149, 4527.051)	4.796 (4.313, 5.146)	0.872 (0.672, 1.072)	30115.402 (21398.446, 44297.824)	16.194 (11.543, 23.722)	97814.446 (75696.128, 134362.616)	20.109 (15.540, 28.244)	0.954 (0.809, 1.100)
	Central Latin America	3384.219 (2931.867, 3876.354)	3.607 (3.143, 4.103)	12172.268 (10942.782, 13423.359)	4.809 (4.325, 5.293)	0.947 (0.860, 1.034)	1119.928 (1063.974, 1191.023)	1.445 (1.361, 1.540)	6475.431 (5836.753, 7126.255)	2.687 (2.423, 2.958)	2.188 (1.963, 2.414)	33622.547 (31641.733, 35883.899)	35.892 (33.808, 38.252)	158077.204 (143505.432, 173854.760)	62.838 (57.108, 69.096)	1.918 (1.720, 2.116)
	Southern Sub-Saharan Africa	1380.597 (1182.359, 1597.132)	4.678 (4.002, 5.361)	2577.128 (2241.390, 2928.288)	4.210 (3.686, 4.730)	−0.518 (−0.683, -0.353)	493.339 (261.567, 751.918)	1.790 (1.660, 1.863)	986.051 (677.238, 1308.935)	3.443 (3.046, 3.693)	−0.339 (−0.592, -0.085)	13659.450 (8255.803, 18871.105)	47.133 (27.177, 67.434)	26228.265 (18454.683, 34420.397)	45.014 (31.731, 59.127)	−0.317 (−0.563, -0.070)
	Middle SDI	30614.740 (26132.251, 35606.257)	2.710 (2.350, 3.096)	89561.470 (79276.365, 100011.022)	3.365 (2.998, 3.731)	0.913 (0.803, 1.023)	8992.623 (7003.998, 12322.476)	1.072 (0.850, 1.453)	33341.760 (27822.479, 40427.277)	1.388 (1.155, 1.680)	1.150 (1.025, 1.275)	269855.735 (212977.030, 366218.845)	25.076 (19.938, 33.571)	812056.090 (695675.277, 984261.992)	30.999 (26.553, 37.412)	0.835 (0.755, 0.914)
	Low SDI	7696.018 (6515.902, 8957.934)	3.185 (2.736, 3.644)	18292.088 (16088.698, 20678.402)	3.325 (2.949, 3.698)	0.156 (0.127, 0.185)	4477.690 (2158.181, 6437.856)	2.342 (1.192, 3.266)	11083.579 (6589.044, 15793.986)	2.610 (1.558, 3.746)	0.632 (0.445, 0.820)	126670.875 (60835.325, 188619.302)	53.162 (27.074, 75.277)	291854.540 (178639.623, 406439.755)	56.021 (34.429, 78.826)	0.313 (0.203, 0.423)
	High SDI	65228.932 (56924.960, 73998.173)	6.202 (5.419, 7.030)	155237.812 (137458.203, 174122.240)	8.187 (7.286, 9.066)	0.919 (0.793, 1.046)	20063.973 (18621.718, 20871.044)	0.640 (0.440, 1.004)	81732.297 (71243.835, 88091.879)	0.763 (0.561, 1.152)	2.302 (2.045, 2.559)	506204.639 (470841.187, 546440.487)	46.538 (43.285, 50.266)	1500929.597 (1354746.322, 1614665.297)	71.397 (65.270, 76.565)	1.537 (1.327, 1.748)

#### 3.1.1 ILD burden based on geographical differences

From a regional perspective, in 2021 (Table 1; Figure 1), Southern Latin America had the highest ASIR (9.904 per 100,000), while Eastern Europe had the lowest ASIR (1.040 per 100,000). The number of patients in South Asia is the highest, reaching 96,281 people, while the number of patients in Oceania is only 419 people, the lowest. From 1990 to 2021, Austrasia region showing the highest upward trend (EAPC = 2.150), while the Eastern Europe region has the largest decrease (EAPC = −2.563). The ASMR is highest in Southern Latin America (4.796 per 100,000), and lowest in Southeast Asia (0.330 per 100,000); the highest number of deaths occurred in South Asia, with 54,724 people, while Oceania had the lowest number of deaths, with only 185 people. The Western Europe region has the largest increase (EAPC = 3.742). The ASDR is highest in the Andean Latin America region (209.339 per 100,000), and lowest in the Southeast Asia region (8.930 per 100,000); The South Asia region has the highest number of DALYs, reaching 1,312,643 people, while the Oceania region has the lowest number, only 8,024 people. Over the past 30 years, the largest increase of ASDR observed in Australasia (EAPC = 3.096).

**Figure 2:**
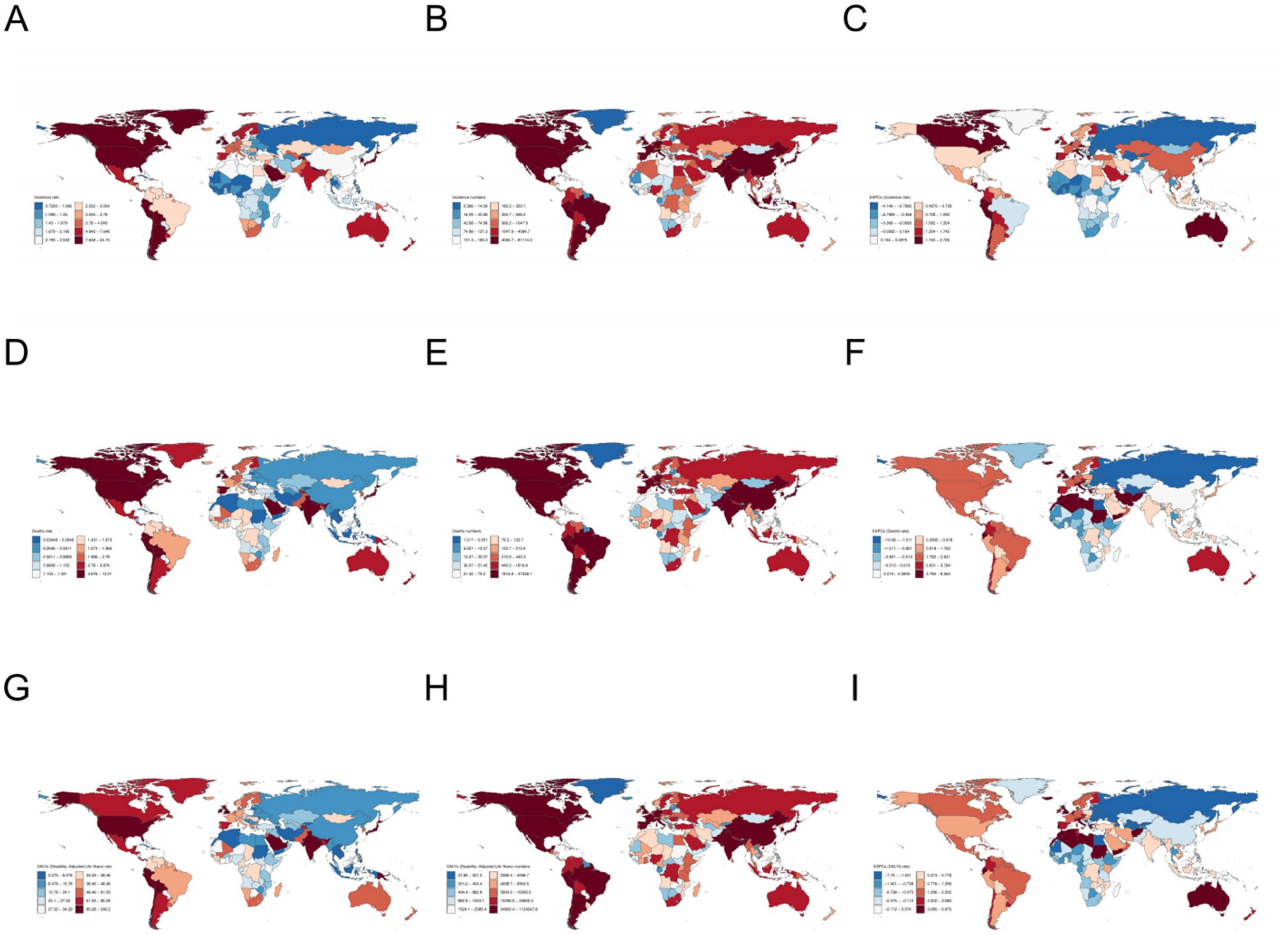
Global map of ILD. **(A–C)** The number of incidence, ASIR, and EAPC of ASIR; **(D–F)** Deaths, ASMR, EAPC of ASMR; **(G–I)** DALY number, ASDR, EAPC of ASDR.

In 2021, Peru, Bolivia, and Chile had the highest ASIR among all countries, with ASIR of 24.725 per 100,000 in Peru, 18.483 per 100,000 in Bolivia, and 15.128 per 100,000 in Chile, respectively. Taiwan (EAPC = 2.860), Ecuador (EAPC = 2.725), and Hellenic (EAPC = 2.722) showed the highest increase in ASIR (Supplementary Table S2; Figure 2). Peru, Bolivia, and Mauritius have the highest ASMR, with Peru having 13.315 per 100,000, Bolivia having 9.743 per 100,000, and Mauritius having 9.574 per 100,000, respectively. Italy (EAPC = 8.564), Libya (EAPC = 8.206), and Morocco (EAPC = 7.166) have the highest ASMR increases. Peru, Mauritius, and Bolivia have the highest ASDR, with Peru having 246.210 per 100,000, Mauritius having 200.518 per 100,000, and Bolivia having 184.192 per 100,000, respectively. Italy (EAPC = 5.973), Saint Vincent and the Grenadines (EAPC = 5.672), and Hellenic (EAPC = 5.301) showed the largest increase in ASDR. At the same time, India, America, and China had the highest number of incidence, with 81,114 people, 59,754 people, and 48,513 people, respectively. India, America, and Japan rank among the top three in terms of deaths and DALY numbers globally, with 47,336 people and 1,124,247 people in India respectively; America has 26,601 people and 524,808 people respectively; Japan has 24,025 people and 383,903 people, respectively. On the contrary, the countries with the lowest ASIR are Philippines at 0.728 per 100,000, Cabo Verde at 0.808 per 100,000, and Burkina Faso at 0.813 per 100,000. The number of incidence is less than one in Tokelau、Niue and Nauru. Ukraine (EAPC = -4.146), Belarus (EAPC = -3.342), and Turkey (EAPC = -2.014) showed the largest decrease in ASIR. The countries with the lowest ASMR are Moldova at 0.039 per 100,000, Iran at 0.046 per 100,000, and Philippines at 0.048 per 100,000. Tokelau, Niue and Nauru had the lowest number of deaths, less than one. Moldova (EAPC = −10.078), Latvia (EAPC = −9.224), and Estonia (EAPC = −8.828) showed the greatest reduction in ASMR. The countries with the lowest ASDR are Philippines with 2.075 per 100,000, Iran with 3.001 per 100,000, and Moldova with 3.061 per 100,000; Tokelau (1.152 people), Niue (1.411 people), and Nauru (6.127 people) had the lowest number of DALYs. Latvia (EAPC = −7.790), Estonia (EAPC = −7.129), and Lithuania (EAPC = −6.464) showed the greatest reduction in ASDR.

#### 3.1.2 ILD burden based on age and gender

**Figure 3:**
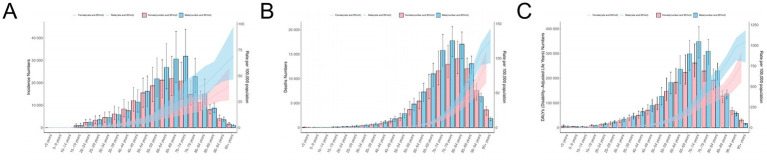
Population analysis of ILD. **(A)** ASIR and the number of incidence; **(B)** ASMR and the number of deaths; **(C)** ASDR and the number of DALYs.

From a gender perspective, in 2021, the number of incidence in male, DALYs, deaths, as well as ASIR, ASMR, and ASDR were all higher than those in females worldwide. In terms of incidence, the number of incidence in male reaches its peak between the ages of 70–74 age group, while the number of female is highest among the 65–69 age group. Both male and females’ ASIR reached the peak in people over 95 years old, and ASIR roughly increased with age. The peak number of males’ deaths and ASMR are between 70 and 75 years old and over 95 years old, respectively, while the peak number of females’ deaths and ASMR are between 80 and 84 years old and over 95 years old, respectively, and the ASMR of females increases with age. The peak values of DALY for both males and females are between 70 and 74 years old, and the peak values of ASDR are above 95 years old (Figure 3).

#### 3.1.3 ILD burden based on SDI

**Figure 4:**
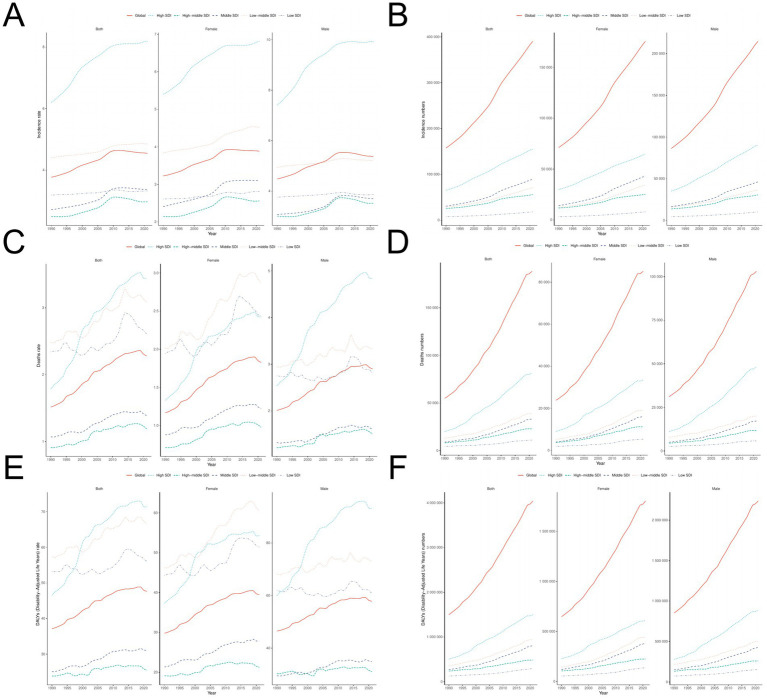
Time trend analysis of ILD. **(A,B)** ASIR and number of patients; **(C,D)** ASMR and death toll; **(E,F)** ASDR and DALY numbers.

From 1990 to 2021, the ASIR, ASMR, and ASDR of the five SDI regions showed a significant upward trend (Figure 4). ASIR in the High SDI region is the highest (8.187 per 100,000), while the ASIR in the High middle SDI region is the lowest (2.970 per 100,000) (Table 1). The number of deaths is highest in the High SDI region (81,732 people) and lowest in the Low SDI region (11,083 people); The ASMR in the High middle SDI region is the lowest (1.192 per 100,000), while the ASMR in the High SDI region is the highest (3.443 per 100,000). The ASDR in the High SDI region is the highest (71.397 per 100,000), while the ASDR in the High middle SDI region is the lowest (25.479 per 100,000); Low SDI has the lowest number of DALYs (291,854 people), while High SDI has the highest number of DALYs (1,500,929 people).

**Figure 5:**
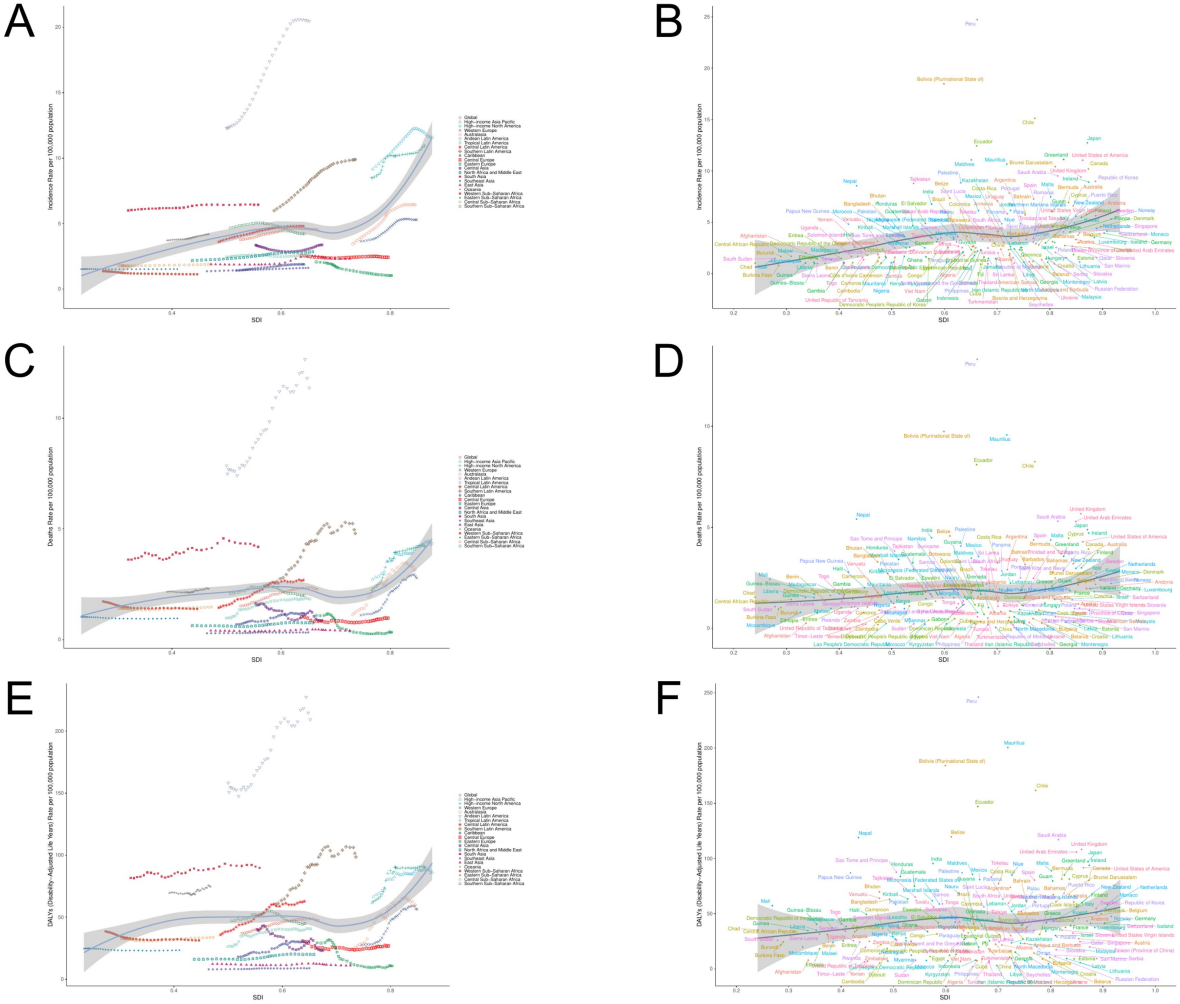
ILD burden and SDI trend analysis. **(A)** ASIR and SDI analysis in region 21; **(B)** ASIR and SDI analysis of 204 countries/regions; **(C)** ASMR and SDI analysis in 21 regions; **(D)** ASMR and SDI analysis of 204 countries/regions; **(E)** ASDR and SDI analysis in 21 regions; **(F)** ASDR and SDI analysis of 204 countries/regions.

For over 30 years (Figure 5), ASIR, ASMR, and ASDR have shown a roughly positive correlation with SDI levels in 21 GBD regions. However, in 204 countries or regions, changes in SDI values have little impact on ASIR, ASMR, and ASDR.

### 3.2 Disease burden analysis of ILD

#### 3.2.1 Joinpoint regression analysis

**Figure 6:**
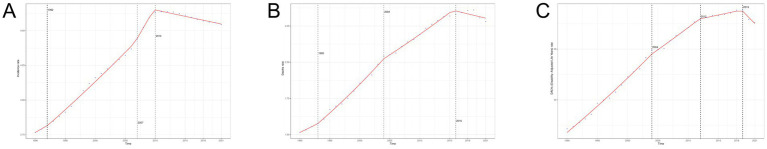
AAPC analysis of ILD. **(A)** ASIR; **(B)** ASMR; **(C)** ASDR.

From the perspective of AAPC (Figure 6; Supplementary Table S3), we divided the period from 1990 to 2021 into four stages. The ASIR of ILD has shown an overall increasing trend since 1990 (AAPC = 0.025), and the period of the largest increase being from 2007 to 2010. The trend of ASIR changes is similar for males (AAPC = 0.028) and females (AAPC = 0.021), with the highest increase in ASIR occurring between 2006 and 2010. On a global scale, ASMR also showed an overall growth trend (AAPC = 0.026), with the largest increase from 1993 to 2004; The trend of changes in male (AAPC = 0.030) and female (AAPC = 0.022;) is similar to that of the overall population, with male ASMR showing the largest increase from 1993 to 2004 and female ASMR showing the largest increase from 1990 to 2004. Both in the overall population (AAPC = 0.350) and in males (AAPC = 0.361) and females (AAPC = 0.319), ASDR showed an overall increasing trend, with the largest increase between 1990 and 2004, but after 2019, all three began to decline.

#### 3.2.2 Decomposition analysis

**Figure 7:**
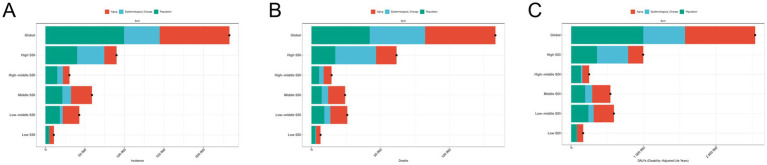
Decomposition analysis of ILD. **(A)** The number of incidence; **(B)** the number of deaths; **(C)** the number of DALYs.

Decomposition analysis shows that the global incidence of ILD is on the rise, with the largest increase occurring in high SDI regions (Figure 7; Supplementary Table S4). The proportion of population aging, population growth, and epidemiological trends in the overall global increase in ILD cases is 37.9, 42.74, and 19.36%, respectively. Among them, aging has a greater impact on males than females, while population growth and epidemiological trends have a greater impact on females than males. Low SDI regions contribute the most to population aging (52.16%), while high SDI regions contribute the most to epidemiological trends (38.12%). The impact of population growth is most pronounced in high middle SDI regions (49.36%). The global death toll is showing a further increasing trend, mainly driven by population growth (31.68%) and aging population (38.18%), with a greater impact on men than women; The relative contribution of epidemiology to its growth is relatively small (30.14%). The middle SDI regions contributes the most to population aging (50.15%), while the low SDI regions contributes the most significantly to population growth (43.2%), and the high SDI regions has the most significant impact on epidemiological trends. In the past 30 years, the number of DALYs caused by ILD has also been increasing, with varying degrees of contribution from population aging (38.13%), population growth (39.18%), and epidemiological trends (22.7%). Among them, population growth and epidemiological trends have a greater impact on women, while aging factors have a more significant impact on men. Low SDI regions have a significant impact on the reduction of DALY numbers due to population aging (51.85%) and population growth (46.96%); In terms of epidemiology, high SDI areas (43.38%) have the highest contribution.

#### 3.2.3 Frontier analysis

**Figure 8:**
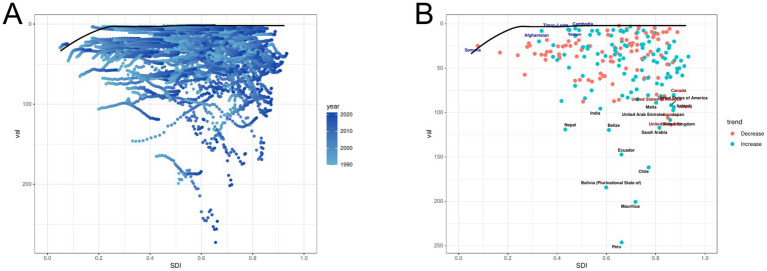
Frontier analysis of ILD. **(A)** Long term trends in disease burden at different SDI levels. **(B)** The gap between countries/regions and frontiers. In Figure A, the color change from light tones (1990) to dark tones (2021) represents the change in year. In Figure B, each point represents a specific country or region in 2021, and the border lines are displayed in black. The top 15 countries and regions with the greatest differences from the border are marked in black. Blue represents the low SDI with the smallest difference from the boundary, while red represents the high SDI with the largest difference from the boundary. The direction of ASDR changes from 1990 to 2021 is represented by the color of the dots, with orange dots indicating a decrease and green dots indicating an increase.

Using data from 1990 to 2021, based on ASDR and SDI, combined with national and regional development levels, conduct cutting-edge analysis to explore potential improvement space for ILD related epidemiological indicators (Figure 8; Supplementary Table S4). The 15 countries and regions with the greatest potential improvement actual differences (effective difference range: 86.892–244.217) include Peru Mauritius, Bolivia, Chile, Ecuador, Belize, Nepal, Saudi Arabia, United Kingdom, United Arab Emirates, Japan, India, Ireland, United States of America and with Malta. The top 10 countries or regions with the smallest difference between ASDR and the frontier line (effective difference range: 0.082–3.638), with countries or regions with lower SDI including Somalia, Timor Leste, Yemen, Cambodia, and Philippines, and countries or regions with higher SDI including Lithuania Montenegro, Moldova, Seychelles, Iran. Frontier analysis reveals potential room for improvement in reducing ILD burden in various countries and regions. Frontier analysis reveals that countries or regions with higher SDI have greater potential for burden improvement.

#### 3.2.4 Analysis of health inequality

**Figure 9:**
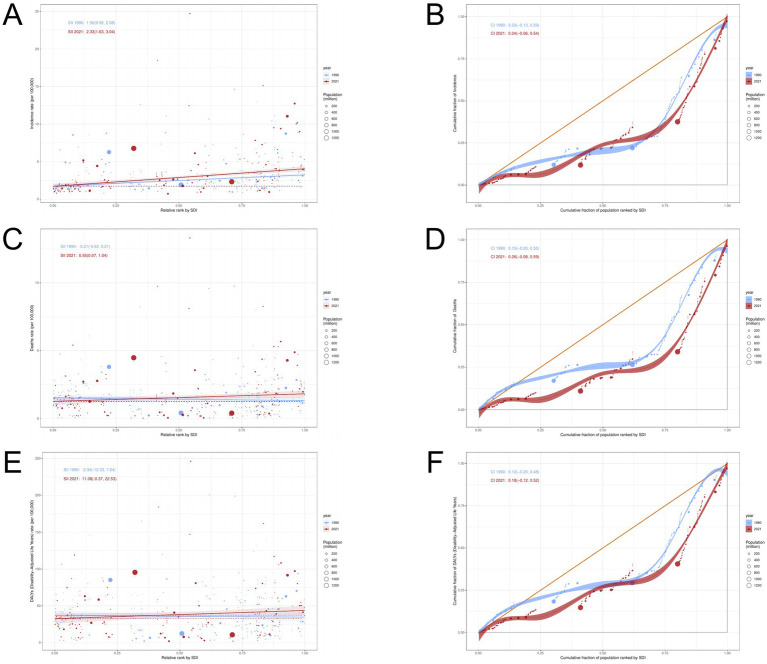
Analysis of Health Inequality in ILD. **(A,B)** ASIR; **(C,D)** ASMR; **(E,F)** ASDR.

This study used SII and CI recommended by WHO to measure transnational health inequalities related to socioeconomic burden in ILD (Figure 9). It can be seen that in terms of ILD ASIR, the SII value increased from 1.499 (95% CI: 0.920, 2.078) in 1990 to 2.334 (95% CI: 1.628, 3.039) in 2021; In terms of ASMR and ASDR, the SII value of ASMR increased from −0.208 (95% CI: −0.625, 0.209) in 1990 to 0.554 (95% CI: 0.068, 1.041) in 2021; The SII value of ASDR increased from −2.344 (95% CI: −12.328, 7.639) in 1990 to 11.083 (95% CI: −0.366, 22.533) in 2021. Countries and regions with high SDI disproportionately bear a heavier burden of disease.

The results of concentration index CI showed that from 1990 to 2021, the ILD incidence rate (1990:0.23; 2021:0.24), Deaths rate (1990:0.15; 2021:0.26) and DALYs rate (1990:0.12; 2021: 0.18) showed different degrees of increase. The above results indicate that there has been no significant improvement in the absolute and relative inequality of ILD disease burden from 1990 to 2021.

#### 3.2.5 Analysis of age-period-cohort effect

**Figure 10:**
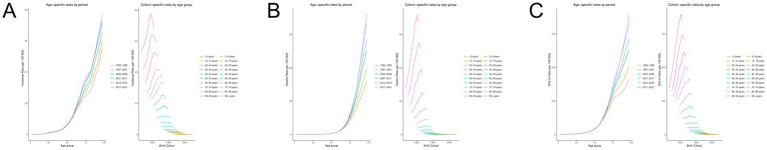
Age specific effects of ILD. **(A)** ASIR; **(B)** ASMR; **(C)** ASDR.

From 1992 to 2021 (Figure 10), the ASIR, ASMR, and ASDR of ILD showed an upward trend with increasing age. The birth queue data shows that the age group over 60 years old shows a trend of first increasing and then decreasing over time, while the remaining age groups remain relatively stable; The age group over 65 years old shows a trend of first increasing and then decreasing over time.

**Figure 11:**
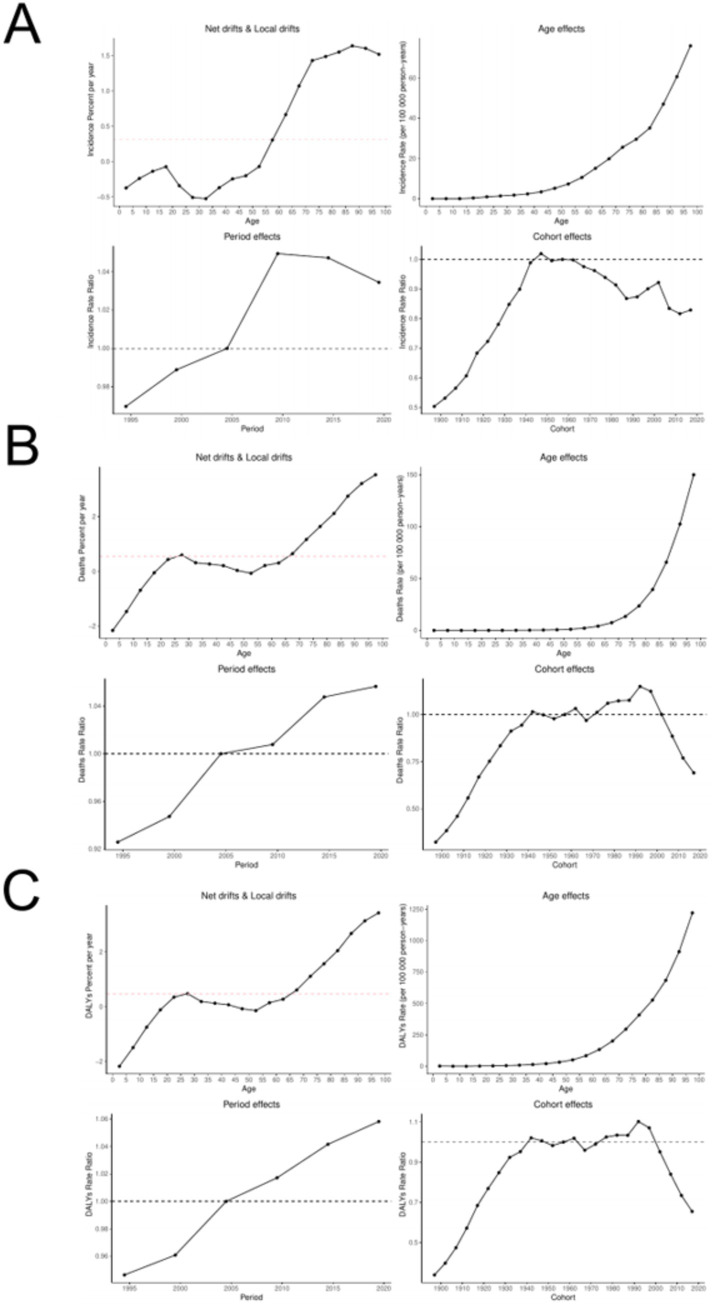
APC model of ILD. **(A)** ASIR; **(B)** ASMR; **(C)** ASDR.

The overall net drift of the ASIR, ASMR, ASDR for ILD was 0.311%, 0.553, and 0.467%, respectively, which reflected that the three had increased to some extent during the study period (Figure 11). The improvement degree of male’ ASIR (0.228% vs. 0.375%), ASMR (0.411% vs. 0.725%), and ASDR (0.356% vs. 0.587%) were lower than that of female. The local drift reflects the additional age changes in the trend related to epidemiological indicators. For ASIR of ILD, the values of the age groups under 54 years old are less than 0, indicating that ASIR of ILD in these age groups has improved, while for ASMR and ASDR, only the mortality of the age groups under 19 years old and the age groups 50–54 years old has improved.

The age effect of ASIR, ASMR, ASDR for ILD shows different trends. With the increase of age, ASIR, ASMR, ASDR for ILD all show an upward trend, and reach the highest value among people over 95 years old. The global time effect shows that throughout the study period, ASIR, ASMR, ASDR for ILD gradually increase over time. Cohort effects show that the risk of developing ILD in individuals born after 2000 has decreased; In terms of mortality and DASMR and ASDR, the population born after 1990 has shown improvement, and the improvement has become more pronounced over time.

### 3.3 Disease burden prediction of ILD

**Figure 12:**
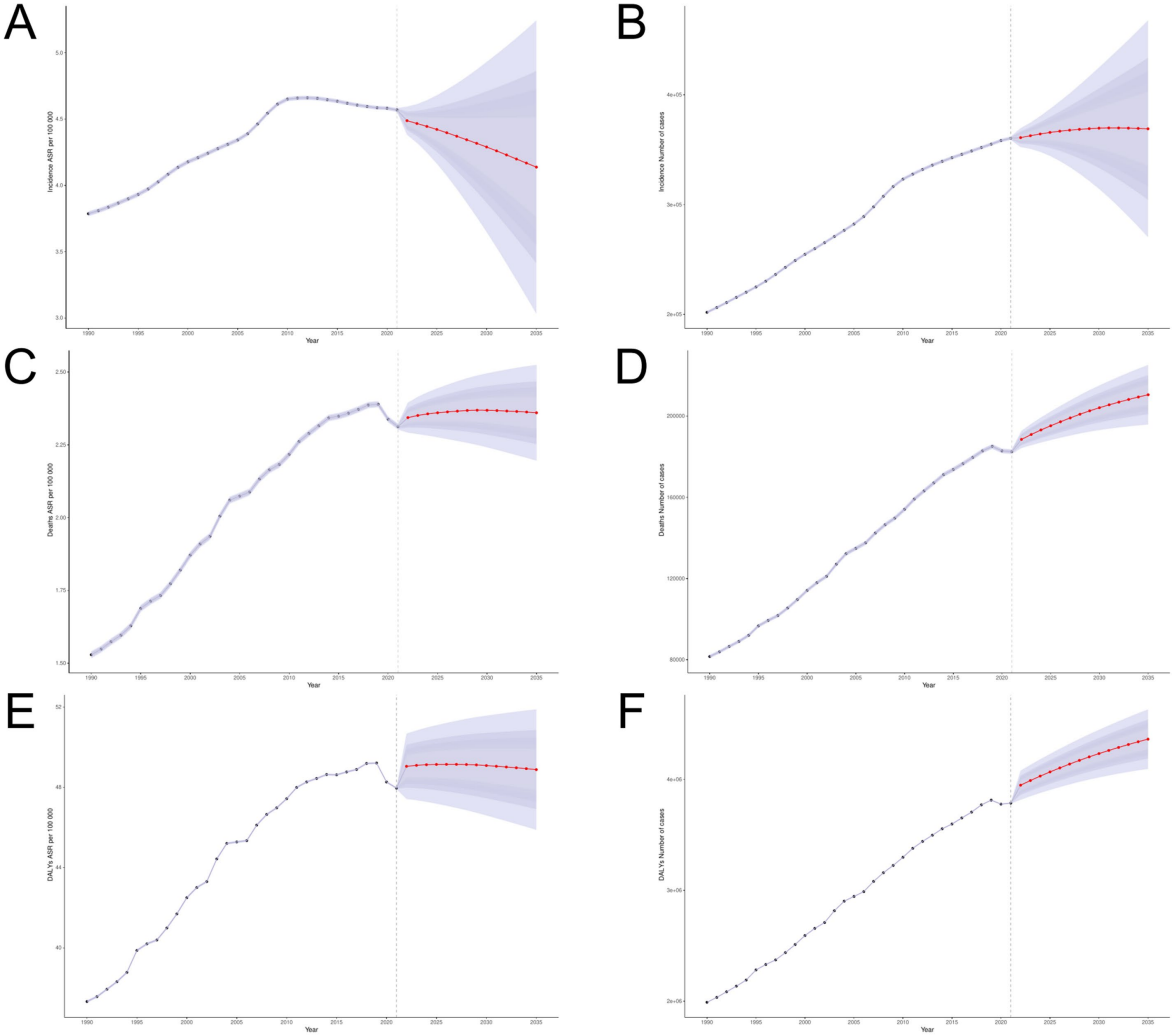
BAPC model. **(A,B)** ASIR and number of incidence; **(C,D)** ASMR and number of deaths; **(E,F)** ASDR and number of DALYs.

**Figure 13:**
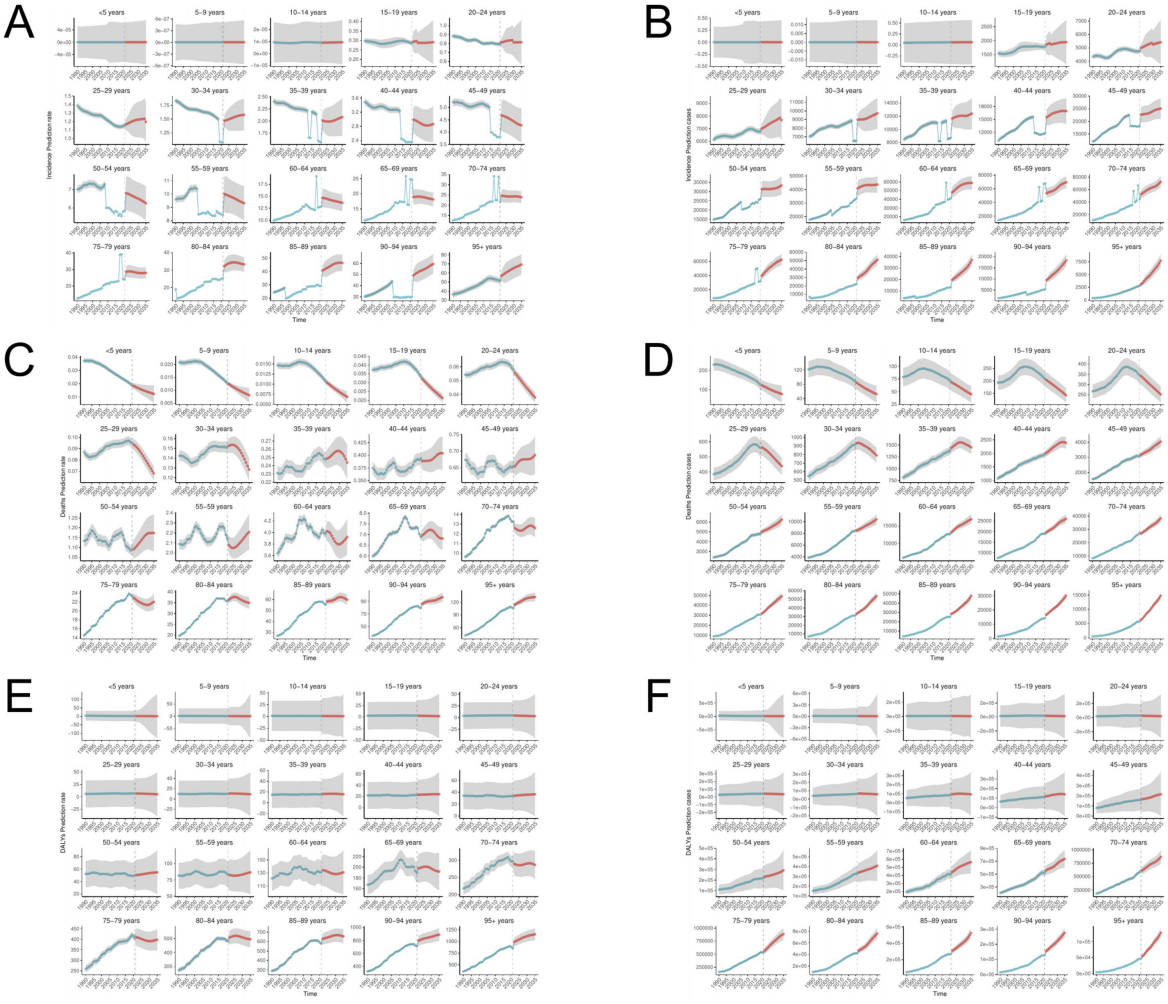
BAPC model for ILD grouped by age. **(A,B)** ASIR and number of incidence; **(C,D)** ASMR and number of deaths; **(E,F)** ASDR and number of DALYs.

BAPC analysis predicted the disease burden of global ILD in 2035 and conducted subgroup analysis in terms of gender and age groups. The number of incidence for ILD is expected to reach 369,120 people by 2035, an increase of 2.38% compared to 2021; The ASIR is expected to reach 4.138 per 100,000, and both male and female ASIRs are on the rise (Figure 12). In all age groups (Figure 13), the number of incidence showed an upward trend in the population aged 20 and above; The trend of ASIR varies, showing an upward trend in the 25–39 age group and the 80 + age group, while a downward trend is observed in the 40–79 age group. The number of death is expected to reach 210,563 people in 2035, an increase of 15.38% from 2021; ASMR is expected to reach 2.360 per 100,000. The ASIR of women remains consistent with the overall trend, showing an upward trend, while the ASIR of men shows a slight downward trend. The number of deaths shows a decreasing trend in the population under 34 years old, an increasing and then decreasing trend in the age group of 35–39 years old, and an increasing trend in the population over 40 years old. The changes in ASMR and the number of deaths generally follow the same trend. By 2035, the number of DALYs is expected to rise to 4,361,257 people, an increase of 15.23% compared to 2021; ASDR is expected to become 48.886 per 100,000, with women’s predicted ASDR consistent with the overall trend, while men’s ASDR shows a slight downward trend. The number of DALY individuals shows a significant upward trend in the age group of 45 and above; ASDR also shows an upward trend in the age group of 45 and above.

## 4 Discussion

ILD is a restrictive and parenchymal lung disease with over 200 causes. According to the latest GBD data, the disease burden of ILD has significantly increased in the past few decades (30). This study conducted a comprehensive assessment of the global burden of ILD and predicted the trend of disease burden by 2035, in order to provide guidance for public health planning and resource allocation.

In the past 30 years, the disease burden of ILD has increased compared to before. This is a phenomenon worth pondering. Firstly, the development of medical imaging technologies such as high-resolution CT and bronchoscopy (31) has greatly improved the accuracy and timeliness of early diagnosis of ILD, enabling more previously undetected or misdiagnosed cases to be diagnosed, resulting in an increase in disease burden in statistical data (32). In addition, with the acceleration of industrialization and urbanization, the problem of air pollution is becoming increasingly serious. Long term exposure to high concentrations of pollutants such as PM2.5 and sulfur dioxide can lead to or exacerbate ILD (33, 34). Meanwhile, occupational exposure, such as inhalation of harmful substances such as asbestos and silica dust, is also an important pathogenic factor (35). Unhealthy lifestyle habits such as smoking and drinking are closely related to the occurrence of ILD. In recent decades, despite the gradual strengthening of tobacco control policies, there are still a large number of people who continue to smoke. In addition, factors such as increased work pressure and lack of sleep may promote the development of ILD (36).

In 2021, the highest values of ASIR, ASMR, and ASDR for ILD were prominently concentrated in Latin American regions such as Peru and Bolivia. Due to the current shortage of medical resources in many Latin American countries, including inadequate medical facilities, shortage of medical staff, and difficulties in accessing basic medical services, coupled with air pollution, water pollution, and other environmental issues, these factors have a negative impact on residents’ health and may lead to higher disease incidence rates (37). At the same time, India ranks first in the world in terms of the number of ILD cases, deaths, and DALY cases. Although India’s healthcare system is constantly improving, there is still a shortage of medical facilities and professional healthcare personnel in some areas, which leads to many ILD patients being diagnosed only when their condition is severe (38, 39). Therefore, the region should strengthen policy coordination and pay attention to the disease burden of ILD (40). It is worth noting that the growth of ASIR, ASMR, and ASDR in some developed countries (such as Australia and Western Europe) has even exceeded the low income, just as in health inequality data, countries or regions with higher SDI disproportionately bear higher disease burdens. Firstly, developed countries are generally facing the problem of population aging, with the elderly population being more susceptible to interstitial lung disease (41, 42). Secondly, developed countries often have more advanced medical technology and diagnostic capabilities, which enables ILD and other diseases to be identified and recorded earlier, leading to an increase in reported incidence rate. This does not necessarily mean an increase in the actual disease burden in the region, but rather reflects an improvement in diagnostic sensitivity and accuracy (43). At the same time, developed countries continue to pay more attention to occupational health and safety, which may lead to an increase in reports and records of occupational diseases, and promote the identification and statistics of ILD and other diseases (44). The disease burden of ILD has also been severely affected during the COVID-19 pandemic, with the global healthcare system experiencing unprecedented overload, exacerbating health inequalities between countries and regions (45).

Our research results show that the burden of ILD is higher in males than in females, and it is recommended to adopt more effective prevention and management measures for males. On the one hand, smoking is one of the important risk factors for interstitial lung disease (9). Men typically have a higher smoking rate than women, which makes them more susceptible to developing smoking related lung diseases. In addition, the duration and quantity of smoking are often higher among males. On the other hand, men’s work in the labor market often involves exposure to harmful substances such as asbestos, chemicals, and dust, which significantly increase the risk of men developing ILD (46, 47). This reflects the importance of gender in disease occurrence and health management. Special attention needs to be paid to male lung health and disease prevention in public health policies and interventions. The age period queue effect shows that the burden of ASIR, ASMR, and ASDR in ILD still exists in older adults, and the risk of disease burden is roughly positively correlated with age, which may be related to the global aging problem (48). This trend suggests that we need to strengthen research on ILD, improve early diagnostic capabilities, implement effective public health policies to reduce risk factors, and implement management and treatment measures for ILD in an aging society.

The cutting-edge analysis in this study is based on the ASR of SDI and ILD, providing key information for the global trend of ILD disease changes from 1990 to 2021. Surprisingly, countries with lower SDI such as Somalia, Timor Leste, Yemen, and Cambodia exist as frontier countries, exhibiting ILD burden results opposite to SDI despite limited socio-economic resources, and they are closer to the ideal benchmark of the frontier. By comparison, some countries with higher SDI (such as the UK, Japan, the US, etc.) have performed poorly in managing the burden of ILD disease and still have significant potential for improvement. This difference indicates that the burden of ILD is diverse under the influence of different healthcare systems. Although SDI is one of the important factors affecting disease trends, other factors such as environment, genetics, and internal conflicts within countries also play a key role in the burden of ILD (49).

We used the BAPC model to predict the global disease burden of ILD, and the results showed that there are still new challenges in the control and management of ILD. Decomposition analysis shows that the increase in the number of ILD patients over the past 30 years is mainly attributed to population expansion, therefore population growth and aging may also be the main reasons for the expected increase in ILD patients by 2035. WHO (50) predicts that the proportion of people over 60 worldwide will increase to 22% by 2050. Therefore, countries around the world should pay attention to this population issue and improve their healthcare systems to fully respond to this demographic change (51).

This study has several limitations. Firstly, our reliance on the 2021 GBD dataset and R software may introduce potential biases and limitations into our analysis. Systematic errors may be introduced during GBD modeling, or the true international disease burden may be underestimated, especially in regions with poor diagnostic capabilities or inconsistent coding, making it difficult to accurately reflect the actual situation in certain areas (52). Crucially, we did not use independent local data for external validation of the GBD estimates. Secondly, the BAPC assumptions may simplify evolving factors such as diagnostic criteria, new therapies (e.g., antifibrotic drugs), and environmental changes. Therefore, predictions based on BAPC may not fully capture potential future inflection points or accelerations/decelerations in disease burden trends (53, 54). Furthermore, the Joinpoint regression assumes a linear relationship between change points, while the decomposition analysis assumes factors to be independent of each other, which may not hold true for complex disease systems such as ILD. It is necessary to conduct a more in-depth reflection on the potential distortions in trend interpretation caused by these assumptions. Thirdly, treating ILD as a single entity obscures key differences in etiology, progression, and prognosis among different subtypes (such as idiopathic pulmonary fibrosis, connective tissue disease-related interstitial lung disease, and hypertensive lung disease). This may lead to an underestimation or misattribution of disease burden trends (55). Finally, the GBD database only controls for certain covariates, implying that unadjusted covariates (such as dietary habits, lifestyle, drug use, metabolic and physiological changes) may affect the study results (56). Future research should prioritize external validation, subtype stratified analysis, and the inclusion of more refined risk factor data.

## 5 Summary

To sum up, ILD, as a diffuse parenchymal lung disease related to a large number of incidence rate and mortality, has caused a considerable disease burden worldwide, and still faces enormous challenges in control and management in the future. For the burden of ILD diseases, corresponding public health policies should be formulated based on gender, age, region, economic development level, etc., to ensure health monitoring and access to medical resources, form an individualized healthcare system, coordinate international cooperation, and narrow the gap in disease burden between countries.

## Data Availability

The original contributions presented in the study are included in the article/Supplementary material, further inquiries can be directed to the corresponding authors.

## Supplementary material

SUPPLEMENTARY Table S1 Checklist of information that should be included in new reports of global health estimates.

SUPPLEMENTARY Table S2 The disease burden data of ILD in countries/territories (1990 to 2021).

SUPPLEMENTARY Table S3 Global and Male/Female AAPC of ILD.

SUPPLEMENTARY Table S4 Decomposition analysis for global, male, female, by SDI regions.

SUPPLEMENTARY Table S5 Frontier analysis of ILD in all countries and regions.
